# Structural Insights into *Bacillus thuringiensis* Cry, Cyt and Parasporin Toxins

**DOI:** 10.3390/toxins6092732

**Published:** 2014-09-16

**Authors:** Chengchen Xu, Bi-Cheng Wang, Ziniu Yu, Ming Sun

**Affiliations:** 1 State Key Laboratory of Agricultural Microbiology, College of Life Science and Technology, Huazhong Agricultural University, Wuhan 430070, China; E-Mail: chenchen.609609@gmail.com; 2 Department of Biochemistry and Molecular Biology, University of Georgia, Athens, GA 30602, USA; E-Mail: wang@bcl1.bmb.uga.edu

**Keywords:** *Bacillus thuringiensis*, Cry toxin, Cyt toxin, parasporin, pore-forming toxins

## Abstract

Since the first X-ray structure of Cry3Aa was revealed in 1991, numerous structures of *B. thuringiensis* toxins have been determined and published. In recent years, functional studies on the mode of action and resistance mechanism have been proposed, which notably promoted the developments of biological insecticides and insect-resistant transgenic crops. With the exploration of known pore-forming toxins (PFTs) structures, similarities between PFTs and *B. thuringiensis* toxins have provided great insights into receptor binding interactions and conformational changes from water-soluble to membrane pore-forming state of *B. thuringiensis* toxins. This review mainly focuses on the latest discoveries of the toxin working mechanism, with the emphasis on structural related progress. Based on the structural features, *B. thuringiensis* Cry, Cyt and parasporin toxins could be divided into three categories: three-domain type α-PFTs, Cyt toxin type β-PFTs and aerolysin type β-PFTs. Structures from each group are elucidated and discussed in relation to the latest data, respectively.

## 1. Introduction

*Bacillus thuringiensis*, as a Gram-positive soil bacterium, is a member of the *B. cereus* group that includes five other species: *B. cereus*, *B. anthracis*, *B. mycoides*, *B. pseudomycoides*, and *B. weihenstephanensi*. It produces parasporal crystal toxins of insecticidal properties during sporulation, which could be distinguished from other species [1]. With significant but specific toxicity, *B. thuringiensis* toxins have been demonstrated against many typical insect orders, such as Lepidoptera (butterflies and moths), Diptera (flies and mosquitoes) and Coleoptera (beetles and weevils). Newly emerging toxins have presented their toxicities towards other insect orders of Hymenoptera, Orthoptera, Hemiptera, Isoptera, Mallophaga, Thisanoptera, *etc.*, and some pests, such as nematodes and mites [2]. These susceptible targets include major invertebrate plant pests from agricultural and forests, as well as some insect vectors of mammalian pathogens [3]. Today, a large number of *B. thuringiensis* strains have been isolated and classified into at least 71 H serotypes by their flagellar immunological reactions [4,5]. Meanwhile, based on the homology of amino acid sequence, more than 300 holotypes of *B. thuringiensis* toxins have been distinguished and categorized into 73 *cry* and 3 *cyt* families [6,7].

During sporulation phase, *B. thuringiensis* produces two types of Crystal (Cry) and Cytolytic (Cyt) parasporal toxins: [2]. Cry toxin is specifically toxic to the majority of insect orders; some Cry toxins have an insecticidal spectrum spanning two or three orders [8,9]. Cry toxin is described as: a parasporal inclusion (Crystal) protein from *B. thuringiensis* that exhibits toxic effects to a target organism, or any protein that has obvious sequence similarity to a known Cry toxin [10]. Cyt toxin shows limited sequence homology to the Cry family. It is toxic to the dipteran larva *in vivo*, but exhibits a broad range of cytotoxicity against dipteran and mammalian cells *in vitro* [11]. Cyt toxin is taken as: a parasporal inclusion (Crystal) protein from *B. thuringiensis* that exhibits hemolytic activity, or any protein that has obvious sequence similarity to a known Cyt protein [10].

Parasporin, which was isolated from non-insecticidal inclusion proteins of *B. thuringiensis*, has been demonstrated with strong cytocidal activity against human cancer cells [12]. Although at first parasporin was distinguished as Cry category, its unexpected remarkable cytotoxicity and increasing members made it unique. In 2006, the Committee of Parasporin Classification and Nomenclature was organized and defined the term ‘parasporin’ as “*B. thuringiensis* and related bacterial parasporal proteins that are non-hemolytic but capable of preferentially killing cancer cells” [13].

In addition, similar to parasporin, binary-like (Bin-like) and mosquitocidal-like (Mtx-like) toxins are related to Bin and Mtx toxins from *L. sphaericus* [14]. Also, the vegetative insecticidal protein (Vip) expressed before the stage of sporulation, represents a novel group of insecticidal toxin [15].

When digested by susceptible insect larvae, *B. thuringiensis* protoxin would be solubilized in alkaline gut environment and proteolytically cleaved by gut proteases [16]. After binding to the specific receptors on the brush border membrane of the midgut epithelium, the activated toxin would lead to insect death and allow spores to germinate [2]. Over the past few years, considerable efforts have been directed towards binding to toxin receptors, toxin structures and the mechanism of pore formation. Two models have been proposed: the pore formation model and the signaling pathway model [17,18]. However, because of lacking adequate evidences, several fundamental gaps—specific receptor interactions and the structure of transmembrane pores—are still beyond our understanding. On the other hand, with the rapid growth of commercialized insecticides and transgenic crops, insect resistance has become the primary threat to the application of *B. thuringiensis* toxins. To overcome the resistance, several strategies have been provided, including modifying receptor binding sites at the corresponding binding region or alternating receptor binding affinities by swapping domains within certain Cry toxins [19]. Research on *B. thuringiensis* toxin structures has contributed to and will continue to focus on the understanding of working mechanism and the development of long-term application in pest management.

*B. thuringiensis* toxins and pore-forming toxins (PFTs) share many common structural and functional features. PFTs is a major category of membrane-damaging toxins, which also could penetrate target cell membrane in a water-soluble form and generate pores, eventually leading to cell death [20,21]. By their transmembrane structure, PFTs are classified as two main groups: α-pore-forming toxins (α-PFTs) and β-pore-forming toxins (β-PFTs). The α-PFTs are predicted to form pores as a bundle of α-helices, which includes colicins, exotoxin A, diphtheria toxins and so on. The β-PFTs, including aerolysin, epsilon, cholesterol-dependent cytolysin, α-hemolysin, *etc.*, are predicted to form β-barrel transmembrane pores [22]. In this review, according to the similarities of known or predicted structures between *B. thuringiensis* toxins and PFTs, we describe Cry, Cyt and parasporin toxins as three categories based on structural features: three-domain type α-PFTs, Cyt toxin type β-PFTs and aerolysin type β-PFTs. Structural features of these three categories, similarities to the PFTs and structure-related functional progress are elucidated in detail.

## 2. Cry Toxins

### 2.1. Structure of Three-Domain Cry Toxins

The development of techniques in molecular biology, X-ray crystallography and Nuclear Magnetic Resonance (NMR) has revolutionized the understanding of protein structure at atomic level and the elucidation of their functions. By X-ray crystallography, several *B. thuringiensis* Cry toxins have been reported (Table 1) [23,24,25,26,27,28,29,30,31].

**Figure 1:**
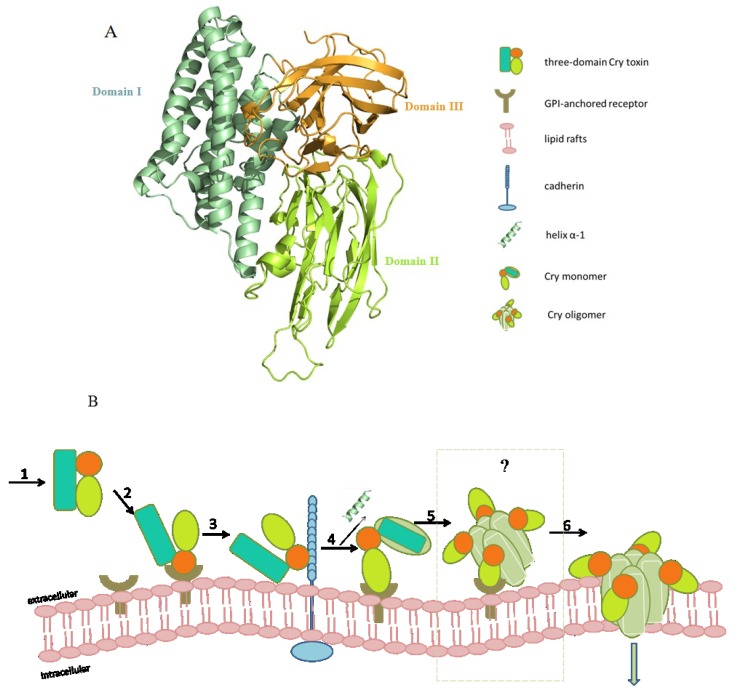
The pore formation model of three-domain Cry1A toxin in the midgut lipid rafts. (**A**) Ribbon diagram of Cry1Aa structure. Three domains are colored in pale green, lemon and bright orange, respectively; (**B**) Sequential steps of the pore formation model. 1. Solubilized Cry1A is digested by the protease in the alkaline insect midgut; 2. Cry1A binds to the abundant GPI-anchored APN and ALP receptors in the lipid rafts with low affinity. This binding promotes the localization and concentration of the activated toxins; 3. Binding to the cadherin receptor facilitates the proteolytic cleavage of the helix α1 at N-terminal end; 4. and 5. N-terminal cleavage induces the formation of pre-pore oligomer and increases the oligomer binding affinity to GPI-anchored APN and ALP receptors; 6. Oligomer inserts into the membrane, leading to pore-formation and cell lysis.

These elucidated structures of Cry toxins exhibit various specific insecticidal toxicities against insect orders of Dipterta, Lepidoptera, Coleoptera, and nematode, which obviously are sharing similar three-domain architectures (Figure 1A). Domain I is an α-helix bundle and shows structural similarity with the pore-forming domain of colicin. It is proposed to be the pore-forming determinant when penetrating the membrane. Domain II is a β-prism of three antiparallel β-sheets packing together; two of them are in a typical ‘Greek-key’ topology and composed of four β-strands. It is suggested to be related to receptor binding. Domain III has a “jelly-roll” topology with the strands forming into a β-sandwich and is considered to be related to receptor recognition and membrane insertion [32]. Domain II and domain III reside at the same side of domain I, stacking against each other along helix α7 [23].

**Table 1:** List of *B. thuringiensis* three-domain Cry toxin structures solved by X-ray crystallography.

Toxin	Target Insects	Expressing Strain	Activation	Resolution	PDB ID	Domain I	Domain II	Domain III	References
Cry1Aa	Lepidoptera	Bt *kurstaki* HD-1	Trypsin 65 kDa	2.25 Å	1CIY	resi 33–253	resi 265–461	resi 463–609, resi 254264	[23]
Cry1Ac *	Lepidoptera	Bt kurstaki HD73	Trypsin 65 kDa	2.35 Å	4ARX	resi 31–263	resi 255–462	resi 463–609	[24]
Cry2Aa	Diptera Lepidoptera	Bt *kurstaki* HD-1	Protoxin 62 kDa	2.2 Å	1I5P	resi 1–272	resi 273–473	resi 474–633	[25]
Cry3Aa	Coleoptera	Bt *tenebrionis* 1911	Papain 67 kDa	2.5 Å	1DLC	resi 61–290	resi 291–500	resi 501–644	[26]
Cry3Bb	Coleoptera	*E. coli* EG7231	67.2 kDa	2.4Å	1JI6	resi 64–294	resi 295–503	resi 504–652	[27]
Cry4Aa	Diptera	Bt *israelensis*	Trypsin 65 kDa	2.8 Å	2C9K	resi 68–321	resi 322–524	resi 525–679	[28]
Cry4Ba	Diptera	Bt *israelensis*	Chymotrypsin 68 kDa	1.75 Å	1W99	resi 84–282	resi 283–466	resi 467–641	[29]
Cry8Ea *	Coleoptera	Bt185	Chymotrypsin 66.2 kDa	2.2 Å	3EB7	resi 64–290	resi 291–500	resi 501–652	[30]
Cry5B	Nematode	Bt *kurstaki* HD-1(Native) *E.coli* M15(Sel-Met)	Elastase 66.14 kDa	2.3 Å	4D8M	resi 112–328	resi 341–541	resi 541–698	[31]

* Three domain regions are estimated according to PDB results.

Complete amino acid sequence alignment of the three-domain Cry toxins reveals that most of the toxins contain five conserved blocks [2]. Conserved block 1 is in the central helix of domain I, block 2 at the domain I and domain II interface, block 3 at the boundary between domains II and domain III, block 4 in the central β-strand of domain III and block 5 at the end of domain III [32].

#### 2.1.1. Domain I of the Three-Domain Cry Toxins

**Figure 2:**
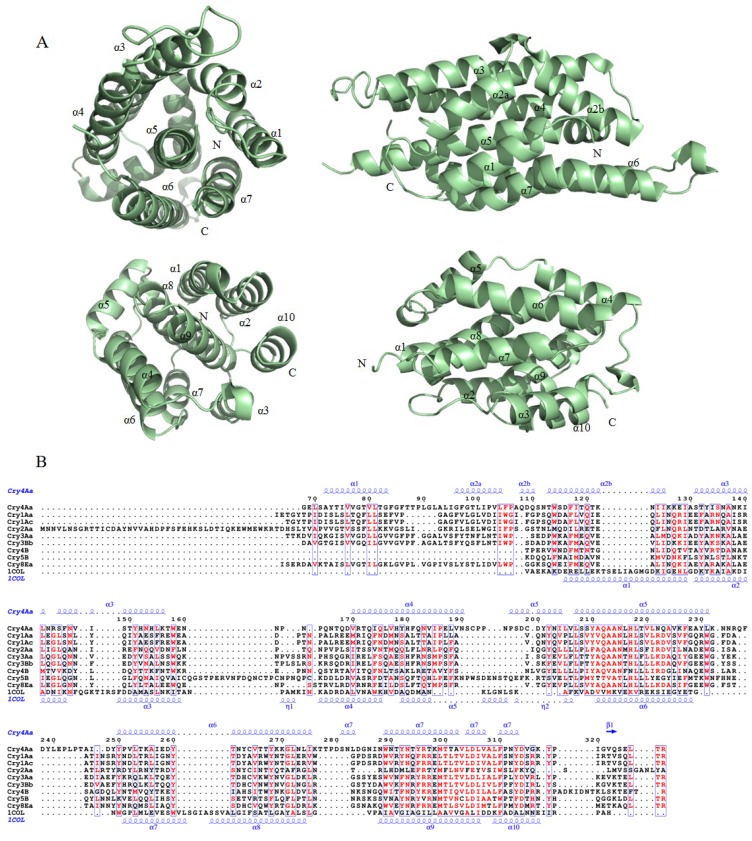
Structural comparison and sequence alignment of domain I of all structure solved three-domain Cry toxins and the pore-forming domain of colicin A (PDB ID 1COL). (**A**) Ribbon diagram of domain I of Cry4Aa (top) and the pore-forming domain of colicin A (bottom); (**B**) Multiple sequence alignment of Cry toxins and colicin A. Secondary structure elements α-helices, 3_10_-helices and β-strands are denoted in blue as α, η and β, respectively. Figure was prepared by ESPript [33].

By structural alignment, domain I of Cry toxin shares a significant structural similarity with the pore-formation domain of α-PFTs colicin A (Figure 2A). Therefore, it has been estimated that domain I might play an essential role in membrane penetration and pore formation after binding to the specific receptors [23]. In Cry3Aa, domain I consists of six amphipathic helices (α1, α2, α3, α4, α6 and α7), which surround the hydrophobic central helix α5 [26]. Helix α2 is interrupted by a short loop section, consequently divided into helices α2a and α2b. In Cry1Aa, Cry2Aa, Cry4Aa, and Cry8Ea, helix α2 is also made up of two short α-helices that are separated by a loop [23,25,30].

After proteolytic digestion, interactions between activated Cry toxins and receptors may facilitate the cleavage of helix α1, and then promote oligomerization. However, recent data have identified that without cadherin receptor, two helix α1 deleted Cry1AbMod and Cry1AcMod mutants could form oligomers by themselves and still remained toxic towards cadherin-silenced *M. sexta* and Bt-resistant *P. gossypiella* [34]. Moreover, the transgenic Cry1AbMod mutant was also toxic to the cadherin-silenced susceptible or tolerant *M. sexta* larvae [35]. Evidence from these examples provides not only a different point of view about the relationship of N-terminal cleavage and oligomerization, but also a potential strategy to counter *B. thuringiensis* resistance in transgenic crops.

As the conserved block 1 covers the central helix α5 and block 2 is corresponding to the C-terminal helix α7, these regions together with their surrounding areas may play a role in its action [26]. Mutations in the helix α4 of Cry1Aa, Cry1Ac and Cry1Ab5 toxins decreased the pore-forming ability, providing the evidence that helix α4 may be involved in pore formation [36,37]. In Cry4Ba, site-directed mutations of two polar amino acids (Asn166 and Try170) at loop α4-α5 almost completely depleted the activity against *A. aegypti* larvae [38]. Similar evidence can also be found at residue Tyr202 of Cry4Aa within the same region [39]. Based on the features of domain I, an umbrella-like model was put forward. In this model, the hydrophobic helices α4 and α5 penetrates the bilayer as an antiparallel hairpin, while the remaining helices spread on the membrane out-surface with a conformation change [40]. The umbrella-like model exhibits the importance of hairpin α4-α5 as the hydrophobic core of domain I and displays an affinitive interaction of membrane penetration and pore formation in the lipid bilayer.

In the umbrella-like model, helix α7 shows a “semi-inserted” orientation and interacts with other helices, which may act as the binding sensor [40,41]. Site-mutagenesis within block 2 of Cry3A has suggested that helix α7 might also be necessary for structure stability [42]. In Cry4Ba, mutations at aromatic resides Trp243, Tyr249 and Phe246 within helix α7 displayed an obvious decrease in toxicities, while Y249 and F264L mutants stay structurally stable upon trypsin activation [43]. Although the hydrophobic core of α4-α5 helical hairpin is the key component of pore-formation, the working mechanism of domain I would be contributed by the collaborations of all the helices.

#### 2.1.2. Domain II of Three-Domain Cry Toxins

**Figure 3:**
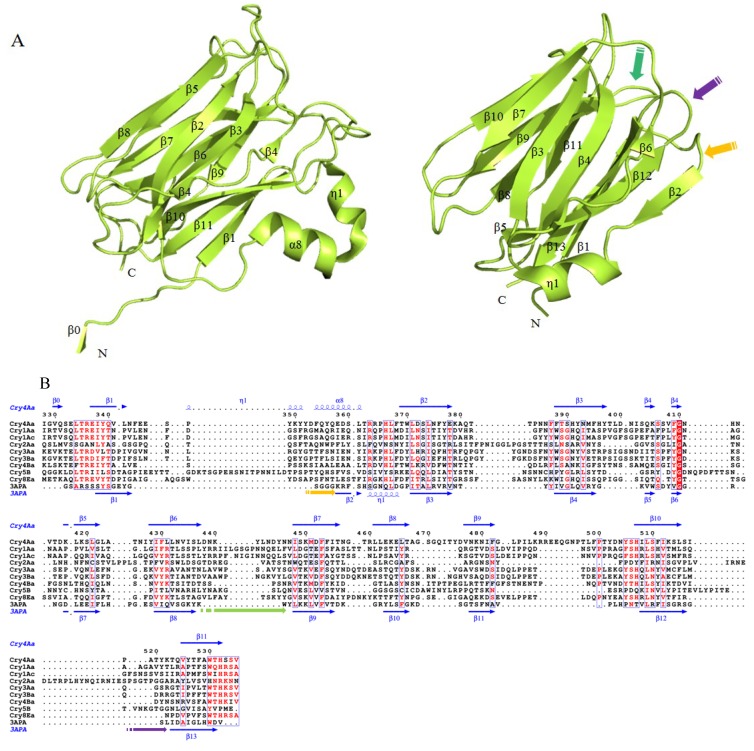
Structural comparison and sequence alignment between domain II of three-domain Cry toxins and ZG16p protein (PDB ID 3APA). (**A**) Ribbon diagram of domain II of Cry4Aa (left) and ZG16p (right); (**B**) multiple sequence alignment of Cry toxins and ZG16p protein. Secondary structure elements α-helices, 3_10_-helices and β-strands are denoted in blue and present as α, η and β, respectively. In ZG16p protein, putative sugar-binding involved GG loop, recognition loop, and binding loop are marked in orange, green, and purple, respectively.

Domain II is a β-prism of three antiparallel β-sheets and two short helices, with a hydrophobic core buried inside (Figure 3A). In Cry4Ba, sheet 1 composed of strands β5, β2, β3, and β4 and sheet 2 consisting of strands β8, β7, β6, and β9, are both in a ‘Greek-key’ motif. The third three-stranded sheet is formed by two separate fragments: C-terminal stands β10 and β11; N-terminal strand β1 and helix α8 [29]. By sequence homology, domain II shows the largest divergence, while domain III presents the highest similarity [29]. As the central part of the β-stands superimposes well, the primary difference is mainly found at the connecting loops.

Loop residues have been targeted by site-directed mutagenesis and membrane binding assays to investigate the molecular basis of insecticidal ability [44]. Binding affinities between mutated loops and receptors have provided an elaborate explanation to the roles of loop β6-β7, loop β10-β11 and loop α8 [45,46]. In Cry1Ab, substitution of loop β6-β7 residue Asn372 increased the toxicity against *L. dispar* and enhanced binding affinity to the membrane, while by deleting the residue Asn372, toxicity and binding affinity were substantially reduced [47]. Mutants G439A and F440A in Cry1Ab loop β10-β11 lost toxicity and binding affinities against *M. sexta* and *H. virescens*, which provided an example of interaction with the glycosyl-phosphatidylinositol(GPI)-anchored aminopeptidase-N(APN) receptor [48,49]. Later, two mutants in loop β6-β7 revealed that binding activity was related to APN and GPI-anchored alkaline phosphatase (ALP) receptors. Although these two mutagenic monomers were not involved in the interaction with APN or ALP, significant evidence of oligomeric Cry1Ab binding to these two types of receptors was observed [50]. It has been suggested that APN and ALP were the initial receptors that promote the localization of toxin monomers into the midgut lipid raft, after that, ALP and APN would facilitate Cry toxin to form oligomer and insert into the membrane [50]. Besides APN and ALP, loop β6-β7 and loop α8 of Cry1Ab toxin have similar binding abilities to the cadherin-like receptor Bt-R_1_ from *M. sexta* [51]. Recent study has revealed that loop β10-β11 of Cry1Aa was involved in binding to BtR175, a cadherin-like protein from *B. mori* [52]. Also, loop β10-β11 of Cry1Ab toxin bound to the cadherin CR12 fragments in *H. virescens*, while loop α8 and loop β6-β7 bound to *M. sexta* cadherin CR7 and CR11 fragments [51,53]. It is worth pointing out that, in Cry1Ab, loop β10-β11 mutants could significantly affect both the binding of the oligomer with Bt-R_1_ and the monomer with APN in *M. sexta* [54]. Considering APN is predominantly populated in the membrane, it has been suggested that monomeric Cry1Ab binds with high abundance but low affinity to APN before the high affinity with cadherin receptor Bt-R_1_ [54,55]. Therefore, a “Ping-Pong” binding model has been proposed based on binding of Cry1Ab and receptors in *M. sexta*: domain II loop β10-β11 may be first involved with the highly abundant but low affinity APN receptor. In the following, loop β6-β7, loop β10-β11 and loop α8 interact with cadherin receptor Bt-R_1_, and by this interaction, removal of helix α1 promotes the oligomerization. Later, the oligomeric toxin binds to APN and ALP through other regions of the Cry toxin, such as strand β16-β22 in domain III, but the oligomer remains the binding to receptor Bt-R_1_. Finally, residues in loop β10-β11 participate in post-APN binding events, such as membrane insertion [54].

#### 2.1.3. Domain III of Three-Domain Cry Toxins

**Figure 4:**
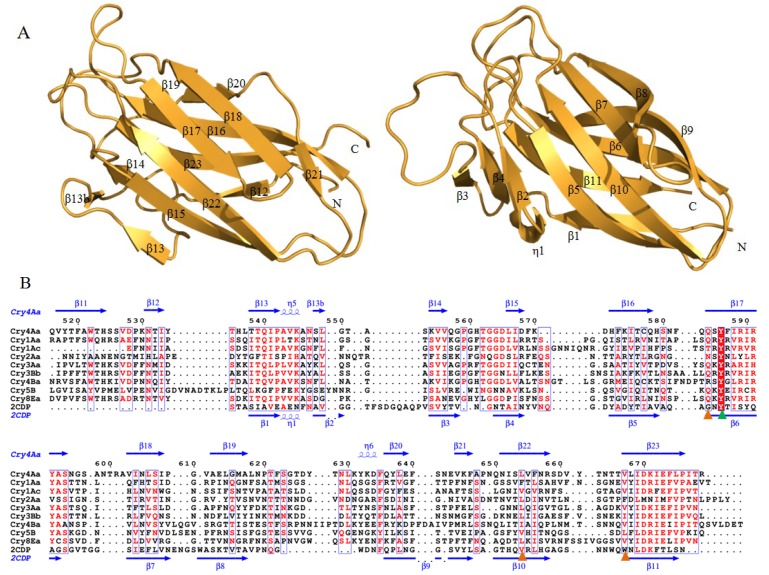
Structural comparison and sequence alignment of domain III of all structural known three-domain Cry toxins and CBM6 family member Aga16B-CBM6-2 binding with neoaragohexaose (PDB ID 2CDP). (**A**) Ribbon diagram of domain III of Cry4Aa (left) and Aga16B-CBM6-2 (right); (**B**) multiple sequence alignment of Cry toxins and Aga16B-CBM6-2. Secondary structure elements α-helices, 3_10_-helices and β-strands are denoted in blue as α, η and β, respectively. Strictly conserved and semi-conserved residues of CBM6 family are marked in green and orange triangle, respectively.

Domain III consists of two twisted anti-parallel β-sheets forming a face-to-face sandwich, with a “jelly-roll” topology [23,26] (Figure 4A). The sheet with the C-terminal strand which contacts with domain I is taken as the inner sheet, while the outer sheet is exposed to the solvent [28]. Although the β-strands or the connecting loops are diverse in lengths or orientations, containing three of the five conserved blocks, the most conserved domain III of Cry toxins also plays an important functional role in toxin action.

The role of domain III has been determined by various mutagenesis or recombination experiments. In *H. virescens*, mutants in Cry1Ac were able to affect receptors’ binding affinities [56]. Hybrid exchanges within domain III also demonstrate the importance of specific interactions with membrane receptors. Experiments of inter-exchanged domain III between Cry1Ab and Cry1Ac or Cry1Aa and Cry1Ac have shown that domain III could be involved in both toxicity and receptor binding activity [57,58]. It was also discovered that Cry1Ac might have two different binding epitopes, while Cry1Aa or Cry1Ab might bind to a single site on APN [59]. Moreover, compared to loop β6-β7 and loop β10-β11 in domain II which are buried inside the oligomeric structure, strand β16-β22 region, especially strand β16, is suggested to be involved in the oligomer binding with APN, but not responsible for pre-pore formation [46]. Mutagenesis of residues Asn506, Gln509 and Tyr513 in strand β16 of Cry1Ac could also reduce the binding to APN in *M. sexta* membrane [60]. A similar observation was found at mutant T542N in loop β16-β17 within the conserved block 4 of Cry1Ac: the insecticidal activity was increased against *S. exigua* larvae [61]. Meanwhile, it was reported that residue Trp544 in Cry1Ac loop β18-β19 has an influence on maintaining toxin stability towards *H. armigera* [62]. Domain III exhibits not only similar functional role as domain II in receptor recognition and binding, but also insecticidal specificity and stability that could be considered as its unique feature. More adequate evidence is still needed.

In order to counter resistance or cross-resistance in transgenic crops, hybrid exchanges of domain III between Cry1C and Cry1Ab has been proven to reduce the cross-resistance in *P. gossypiella* and *P. xylostella*, suggesting that domain III of Cry1C would be required for anti-resistance methods, while domain II of Cry1Ab alone might have limited contribution [63]. As domain II and domain III, as well as domain I of Cry toxins are responsible for the sequential process of activation, receptor binding or oligomerization. More importantly, some particular steps are required for cooperative contributions by different domains. In-depth understanding of the structure–function relationship of Cry toxins would benefit the design of modified toxins and provide potential strategies to counter insect resistance.

#### 2.1.4. Structure of Three-Domain Cry Toxins in Parasporin

In 2005, structure of parasporin-1 from *B. thuringiensis* stain A1190 was determined at 1.76 Å. It was described with limit sequence identity but significant structural similarity to the structure known in three-domain Cry toxins [64]. Of preferential anticancer cytotoxicity, parasporin-1 shows distinct biochemical features towards HeLa (Uterus gland cancer cell) cells: no obvious LDH (lactate dehydrogenase) leakage, limited fluorescence increased intensity, but remarkably, intracellular Ca^2+^ level rising after a few minutes of treatment [65]. It was proposed that instead of forming pores in the membrane, parasporin-1 prefers inducing cell death by an apoptotic pathway [65] (see Section 4.2.1).

Parasporin-3 (PS3Aa1 and PS3Ba1) from *B. thuringiensis* strain A1462 was the first reported toxin with typical three-domain architecture and is cytotoxic to human cancer cells [66]. Sequence alignment shows that parasporin-3 has the same five conserved blocks as the insecticidal three-domain Cry toxins. It has been suggested that parasporin-3 might have a similar mode of action with the three-domain Cry toxins [66]. Recent research has shown that an additional C-terminal β-trefoil ricin domain acted as the putative carbohydrate binding epitopes [67]. Meanwhile, unlike the most diverse regions that existed in domain II of Cry toxins, the C-terminal conserved domain (block 3 to block 5) of PS3Aa1 and PS3BA1 shows the maximum divergence, which may be responsible for the difference in their cytotoxicity preferences [66] (see Section 4.2.3).

Among newly discovered parasporins, parasporin-6 exhibits 56.4% sequence similarity with Cry2 toxin, which suggests that its mechanism of action may be also in accordance with three-domain Cry toxins [68]. Despite the fact that the creditable crystallography evidence of these three-domain-like parasporins is still inadequate, structural and functional related work will provide an unambivalent breakthrough in the *B. thuringiensis* research area.

### 2.2. Comparisons with Other Structure Known Toxins or Modules

Based on the comparison between domain I of Cry toxins and pore-forming domain of α-PFTs, the three-domain Cry toxin was suggested to belong to the group of α-PFTs [69]. The α-PFTs share a number of similarities in their pore-forming activities, which are essentially represented by colicins and *Staphylococcus* δ-toxins (or δ-haemolysins) [22]. Colicins are a family of water-soluble bacterial toxins that span lipid bilayers of *E.* *coli* by forming voltage-dependent channels [70]. One of the colicins, colicin A, has a three-domain architecture: a receptor binding domain that specifically binds to the outer membrane via Vitamin B_12_ receptor; an N-terminal translocation domain that delivers pore-forming domain inserting into the periplasm; and a C-terminal pore-forming domain that forms a voltage-gated channel in the cytoplasmic membrane, that because of this, means it destroys the membrane potential [71,72,73]. The pore-forming domain of colicin A is similar to the domain I of Cry toxin and consists of ten α-helices, with a hydrophobic hairpin (H8 and H9) in the center (Figure 2B) [70]. According to the polarity parameter valves over the helices, it has been indicated that helix α9 was buried inside membrane, while other surrounding helices were lay on the surface. Therefore, like Cry toxin, an umbrella model of colicin A has been concluded: the amphipathic helices α1-α7 and α10 are located on the membrane-water interface and the hydrophobic hairpin helix penetrates the membrane [71].

Structural comparison by Dali server shows a Z-score (a measure of structural similarity) as high as 11.8 between domain II of Cry toxin and the structure of ZG16p protein (PDB ID 3APA), using domain II of Cry4Aa (PDB ID 2C9K) as template [74,75]. ZG16p is a 16 kDa secretory protein from rat pancreatic zymogen granules in cholesterol-glycosphingolipid-enriched microdomains, which was suggested to regulate the binding of aggregated zymogens to the granule membrane [76,77,78]. It has a β-prism fold consisting of three β-sheets, and the overall structure is similar to Jacalin-related mannose-binding-type lectins [74,79]. As the first solved structure of β-prism fold in mammalian lectins, ZG16p is made up of 12 β-strands and arranges into three separated ‘Greek-key’ motifs. The short α-helix between strands β2 and β3 is a unique feature among the β-prism fold lectins, but it also exists in Cry toxins [74]. On the top of the β-prism, three loops are proposed to be involved in sugar-binding activity: GG loop (between β1 and β2), recognition loop (between β8 and β9) and binding loop (β12 and β13), which are similar to the receptor binding regions of loop α8, β6-β7 and β10-β11 of Cry toxins (Figure 3B). The positive-charged lysine and arginine clusters of ZG16p that are close to the putative binding related loops have shown potential interaction with mannose/glucose-type glycans and glycosaminoglycans [74]. By structural comparison with ZG16p or other β-prism fold lectin proteins, more putative sugar-binding sites of three-domain Cry toxins could be found in the future.

When using domain III of Cry4Aa as template, Dali comparison shows a Z-score of 14.7 with Aga16B-CBM6-2, a member of carbohydrate-binding module family 6 (CBM6) from *S. degradans* (PDB ID 2CDP) [75,80]. Carbohydrate-binding module (CBM) recognizes polysaccharides, such as cellulose, chitin, starch, glycogen, xylan, β-glucans, and many other sugars such as mannan, lactose, galactose, β-D-galactosyl-1,4-β-D-N-acetylglucosamine, and blood group A/B antigens [81]. As a branch of CBM, CBM6 mainly binds to cellulose, xylan, mixed β-(1,3)(1,4)-glucan, β-1,3-glucan, and agarase with different binding selectivity. It contains two carbohydrate binding clefts, which may specifically present as binding and recognition sites against its diverse substrates: cleft A locates at the loop that connects the inner and outer β-sheets of the “jelly-roll” fold; cleft B stays on the concave surface of one β-sheet [82]. Structure-based alignment within 26 CBM6 members shows a conserved β-sandwich architecture, but shares little sequence similarity within binding and recognition regions against their various binding substrates. These regions are mainly located at exposed flexible loops or adjacent β-strands. One of the only three strictly conserved residues, the aromatic residue Tyr519, is found in strand β17 of Cry toxins (Figure 4B) [80]. The structure–function relationship of CBM6 family may provide clues to investigate why the three-domain type Cry toxins have various receptor binding specificities and toxicities towards different targets.

### 2.3. Mechanism of the Three-Domain Cry Toxin

Over the past few years, the working mechanism of Cry toxins has continuously been the key topic of research publication and academic discussion. Valuable efforts have focused on gaining a comprehensive understanding of receptor binding and membrane insertion. Based on these findings, two main models of action have been proposed.

#### 2.3.1. The Pore Formation Model

The pore formation model of the three-domain Cry toxins was primarily put forward based on the interaction of Cry1Ab and brush border membrane vesicles (BBMV) of *M. sexta* [18]. This model consists of several sequential steps. Crystal inclusions are firstly digested by the susceptible larvae in alkaline gut environment, where the solubilized inactive proteins are cleaved by midgut proteases at the N or C terminus into an activated protease-resistant three-domain monomer. Before membrane insertion, activated toxin binds to multiple specific receptors located on the surface of midgut membrane. In lepidopteran species such as *M. sexta*, at least two types of specific receptors are involved in binding activity. APN and ALP are supposed as the primary receptors. The activated Cry1Ab toxin first binds to APN and ALP with highly abundant but low affinity binding sites as to promote localization and concentration of the activated toxins in the midgut membrane before interacting with the second receptor of cadherin [50,83]. The interaction with cadherin facilitates proteolytic cleavage of the helix α1 at the N terminus, which induces the formation of a pre-pore oligomer [84]. Later, binding affinity is strengthened by the interactions between oligomers and APN or ALP, which consequently leads to the oligomer insertion into the membrane, causing pore formation and cell lysis (Figure 1B) [54,83,85].

In order to investigate the features of pores in the midgut cell membrane, a sequence of biochemical and biophysical experiments have been taken. However, limited by the challenges of obtaining and testing oligomers from water-soluble state to membrane-bound state, recent work is still lacking an unequivocal understanding of when oligomerization happens, the construction of oligomer, and the size of transmembrane pores. In the pore formation model, monomers oligomerize by sequentially binding to the specific receptors before penetrating the membrane. Whereas, the controversial evidence has questioned that Cry toxins insert into the membrane as monomers, then aggregate and assemble into oligomers within the membrane (Figure 1B). As described, Domain I of three-domain Cry toxins is believed to be required for oligomerization and pore-forming process. The proposed umbrella-like model of domain I shares an analogous model of transmembrane action with colicin A toxin [71]. Recently, the discovery of the colicin Ia toxin with the similar pore-forming domain with colicin A, has suggested a clear evidence of a multimeric state (6–8 monomers) in the lipid bilayer by Cryo-EM and image processing methods. Also, electrophysiological results have indicated a co-existence of monomer, dimer, trimer, even up to hexamer, which supported the hypothesis that colicin Ia may insert into the lipid bilayer as monomer [86]. Similar results were observed in Cry1Aa. A co-existence of monomer, dimer, trimer, and tetramer has been found during the oligomerization process of Cry1Aa, which provided the evidence that Cry1Aa monomer inserts into the lipid bilayer before oligomerization [87].

#### 2.3.2. The Signaling Pathway Model

On the other hand, a cadherin involved alternative model has been put forward. The signaling pathway model proposes that cytotoxicity is mediated by the specific binding with cadherin receptors, which activates a Mg^2+^-dependent cellular signal cascade pathway that leads to the cell death [17]. Moreover, it was suggested that the specific binding to cadherin could stimulate G protein and adenylylcyclase (AC), and then promote cyclic adenosine monophosphate (cAMP) concentration and activate proteins kinase A (PKA), consequently resulting in destabilization of cytoskeleton and ion channel in the membrane [85,88]. The pore formation model is supported by the experimental results from different insect orders, while the signaling pathway model is established on the interaction between Cry1Ab and insect High Five (H5) cell line expressing a cadherin receptor from *M. sexta* [17,89,90]. An earlier study with Sf9 lepidopteran cell line indicated that both plasma membrane permeabilization and intracellular signaling were related to Cry1C binding activity, that provided extra evidence in favor of the signaling pathway model [91]. Besides, a combination of these two models was also suggested based on the action of Cry1Ac against *H. virescens* larvae [90,92]. Signal transduction pathway could possibly be affected or triggered by sequential binding stages of Cry toxins, but it needs more detailed and reasonable evidence and analysis. As an across discipline of biochemistry, crystallography, Cryo-EM, mathematical modeling, and computational biology is emerging nowadays, applications of new technologies may contribute to a comprehensive understanding of the mechanism of action in *B. thuringiensis* toxins.

## 3. Cyt Toxin

*B. thuringiensis* has two main toxin families, insect-specific Cry toxins and cytolytic Cyt toxins. Unlike Cry toxins, Cyt toxins show specific insecticidal activity towards dipteran insects (mosquito and black flies) *in vivo* but a broad range of cytotoxicity against dipteran and mammalian cells *in vitro* [11,93,94]. A distinct group of *B. thuringiensis* toxins produced by *B. thuringiensis* subsp. *israelensis* (Bti) is the main choice for bioinsecticide used worldwide to control mosquitoes and black flies vectors. The toxin-encoding plasmid pBtoxis from Bti has been mapped: it encodes four Cry toxins (Cry4Aa, Cry4Ba, Cry10Aa, and Cry11Aa) and three Cyt toxins (Cyt1Aa, Cyt2Ba and Cyt1Ca) [95]. Due to the functional synthesis of Cyt and Cry toxins, Bti-based commercial product is an efficient method to overcome the resistance in mosquito control.

### 3.1. Structure of the Cyt Toxins

**Figure 5:**
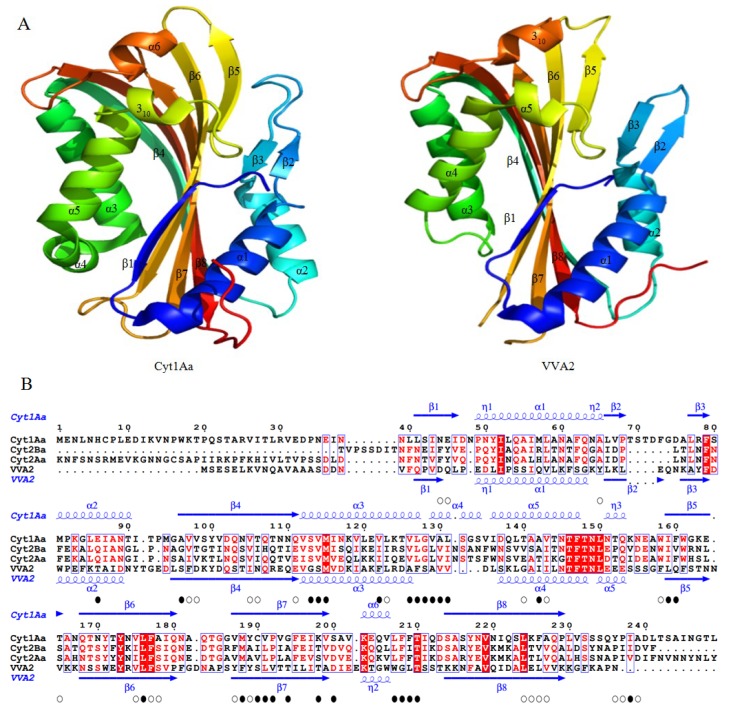
Structural comparison and sequence alignment of Cyt toxins and VVA2 toxin (PDB ID IVCY). (**A**) Ribbon diagram of Cyt1Aa and VVA2 toxins. The overall structure has a single domain of α/β architecture with a central β-sheet surrounded by two α-helical layers. The central β-sheet consists of six antiparallel β-strands with two α-helices on one side and three on the other; (**B**) multiple sequence alignment of Cyt toxins and VVA2 toxin. Secondary structure elements α-helices, 3_10_-helices and β-strands are colored in blue and marked as α, η and β, respectively. The identical or semi-conserved hydrophobic residues in all sequences of the alignment are marked at the bottom with black solid circle and open circle, respectively.

Up until now, the known Cyt subfamily members include Cyt1 (1Aa, 1Ab, 1Ab, 1Ac, and 1Ad), Cyt2 (2Aa, 2Ba, 2Bb, 2Bc, and 2Ca) and Cyt3Aa1. They share a high level of sequence identity [6]. The monomer crystal structures of Cyt1Aa (PDB ID 3RON), Cyt2Aa (PDB ID 1CBY), and Cyt2Ba (PDB ID 2RCI) have been published (Figure 5) [96,97,98]. Structural similarities are observed among these three toxins. The overall structure has a single domain of α/β architecture with a β-sheet in the center surrounded by two α-helical layers. The central β-sheet consists of six antiparallel β-strands, flanked by an α-helix layer composed of α1 and α2 on one side and α3-α5 on the other [96,97,98]. Sequence alignment reveals that there are four blocks with high similarity scores: (I) Block 1, helix α1; (II) Block 2, α5 to β5 region; (III) Block 3, region β6-β7; (IV) Block 4, region α6-β8 [99]. In Cyt1Aa, hairpin β2-β3 between helices α1 and α2 is common to all members of the Cyt1 family, but absent in the Cyt2 group. Hairpin β6-β8 of Cyt1Aa consists of a modified ‘Greek-key’ topology followed by strand β4. By comparing the N terminus of Cyt1Aa and Cyt2Aa, it has been revealed that an extra strand β0 at N-terminal end which only exists in Cyt2Aa may play an important role in dimerization and proteolytic activation [96].

### 3.2. Comparisons with Other Structure Known PFTs

Crystal structures of Cyt toxins demonstrate a significant similarity with volvatoxin 2 (VVA2) (PDB ID 1VCY) (Dali score >20), although primary sequence alignment shows very low identity (less than 17%) (Figure 5) [75,96,97,100,101]. Volvatoxin A (VVA) is a cardiotoxic toxin isolated from edible mushroom *V. volvacea*. It can cause cardiac arrest via activation of the Ca^2+^-dependent ATPase in the ventricular microsomal fraction, resulting in the hemolysis of human red blood cells and cytotoxicity against tumor cells and the mitochondria of liver cells [102,103]. VVA is composed of VVA1 and VVA2, and the latter is a novel pore-forming cardiac toxin related to hemolytic and cytotoxic activity [100,104]. Similar to Cyt1Aa, hairpin β2-β3 of VVA2 inserts within helices α1 and α2, but this hairpin is absent from Cyt2Aa [96,97,100]. In VVA2, the amphipathic helix α2 at N terminus was thought to trigger oligomerization before penetrating the membrane, while residues 128–199 at C terminus (corresponding to β5-β7) may be involved in membrane binding [104]. The proposed VVA2 mode of action would provide valuable insights into the action of Cyt toxins.

### 3.3. Binding Mechanism of the Cyt Toxin

Cyt toxin does not bind to specific receptors located on the midgut epithelial cells as Cry toxin, but directly interacts with saturated membrane lipid such as phophatidylcholine, phosphatidylethanolamine and sphingomyelin [105]. Proteolytic cleavage sites are found at N terminus, while three major β-strands (β5, β6 and β7) at C terminus are probably protected against proteolysis during membrane insertion [106]. Helices α1 and α3 of Cyt1Aa have been suggested to be not only involved in membrane interaction as a specificity determinant but also in intermolecular assembly [107,108]. Meanwhile, by structural comparison to the model of VVA2 that would adopt a molecular packing for pore formation, strands β5, β6, and β7 of Cyt2Aa are long enough to span the hydrophobic region of bilayer and may oligomerize to form membrane-spanning β-barrel [98,100]. It has been postulated that six monomers of Cyt toxins assemble into an ‘open-umbrella’ conformation. Strands β5, β6 and β7 span the lipid bilayer as the handle of an umbrella, while the top of the umbrella is made of the α-helices, splaying on the membrane surface [99]. Therefore, based on the results of the Cyt1Aa working mechanism and crystal structure, a pore-forming model of Cyt toxins has been proposed as follows: Cyt toxin binds to the membrane as a monomer at C-terminal region, then a conformational change at the N terminus triggers oligomerization that leads the β-strands spans the lipid bilayer, resulting in membrane permeabilization [105]. Contrastingly, there is another hypothesis describing that, while Cyt toxin aggregates and splays on the surface of the membrane, it destroys the lipid bilayer in a detergent-like manner [99]. These two models could relate to each other when the concentration of Cyt toxin changes. Cyt toxin may oligomerize at a low concentration, and when toxin/lipid ratio increases to a critical level, membrane could not adapt to a large number of assembled molecules and consequently breaks up into protein/lipid complexes [99].

### 3.4. Mechanism of Synergism with the Cry Toxins

Besides the membrane binding activity, Cyt toxins could also bind to Cry toxins and enhance their specific binding activities to the membrane. The synergism examples were demonstrated between Cyt and Cry11Aa or Cry4Aa from Bti [109,110,111,112]. Two epitopes of Cyt1Aa, _196_EIKVSAVKE_204_ (on helix α6 and strand β7) and _220_NIQSLKFAQ_228_ (on strand β8) are involved in the binding to Cry11Aa [96]. On the other hand, loop α8, strand β4 and loop 2 (residues 386–396) of Cry11Aa are the binding epitopes interacted with Cyt1Aa, which are the same binding regions of Cry11Aa and its receptor [110]. Additionally, two mutants K225A and K198A of Cyt1Aa, which are on the two separate epitopes, had a correlative effect on the oligomerization and pore formation of Cry11Aa [113]. This supports the finding that Cyt1Aa provides binding sites to Cry11Aa and induces the formation of oligomeric structure, which plays a similar role as the cadherin receptor in *M. sexta* [113,114]. Recently, Cyt1Aa mutants K198A (on strand β7), E204A (on helix α6) and K225A (on strand β8) on these two separate epitopes have been proved to be involved in the binding with Cry4Ba, and domain II loop α8 of Cry4Ba is also involved in the synergism with Cyt1Aa [111]. These results suggest that Cyt1Aa acts as a membrane receptor and synergizes with Cry4Aa or Cry11Aa from Bti at similar epitopes, which, to some extent, ensures the insecticidal toxicity of Bti more efficiently than the functional synthesis of other Cry toxins [115,116]. Understanding the mechanism of synergism between Cyt and Bti toxins might be significant to provide strategies for mosquito control, and would also provide valued experience to overcome insects’ resistance to other species.

## 4. Parasporin Toxins

### 4.1. Definition and Classification

Recently, a novel but unique category of *B. thuringiensis* was discovered, and the non-insecticidal parasporal inclusions were related to an unknown biological activity [12,117]. In 1999, non-insecticidal *B. thuringiensis* strain A1190 (former strain 84-HS-1-11) and A1547 (former strain 90-F-45-14) were demonstrated with novel cytocidal toxicities against human cancer cells [117]. These proteins are heterogeneous in both cytotoxicity spectra and activity [12]. Up to date, six subclasses of parasporins have been recorded (PS1, PS2, PS3, PS4, PS5, and PS6), according to the homology of primary sequences by the Committee of Parasporin Classification and Nomenclature [13]. This kind of toxins exhibits strong cytotoxic activities with various toxicity spectra and levels, leading to the morphological changes of intracellular vacuolations, cell swelling and cell bursting [118,119]. Currently, research work centers on the characteristics of putative receptors and the interactions of parasporin with the cell membrane. Crystal structures of the parasporin-2 and the nontoxic 26 kDa protein might be breaking ground and providing fundamental knowledge into receptor recognition and pore-forming mechanism.

### 4.2. Cytotoxicity and Action Mechanism

#### 4.2.1. Parasporin-1

The first discovered parasporin-1 was isolated from *B. thuringiensis* A1190 strain (formerly 84-HS-1-11) [12,120]. In the parasporin-1 group, PS1Aa1 consisted of 15- and 56-kDa fragments and was generated by trypsin activation from 81 kDa pro-PS1Aa1 [117]. These two fragments are tightly associated with each other as an active complex, which could not be separated even in the presence of detergent of denaturing agent. According to structural analysis, the 15- and 56-kDa fragments could be arranged into a heterodimer at a ratio of 1:1 [120]. PS1Aa1 presents strong but specific cytotoxicity towards HeLa, MOLT-4 (Leukemic T cell), HL60 cells (Promyelocytic cell), with the LC_50_ of 0.12, 2.2 and 0.32 μg/mL, respectively. Cell membrane measurement of LDH leakage assay has demonstrated that parasporin-1 did not affect membrane permeability. Also, in HeLa cells, limited changes of fluorescence concentration after being treated with paraspron-1 has shown that parasporin-1 may not form pores in the membrane. On the other site, parasporin-1 could promote Ca^2+^ influx from the extracellular buffer, and the G-protein inhibitor, suramin, can suppress Ca^2+^ influx and the cytotoxicity of parasporin-1 [65,120]. Therefore, it was proposed that instead of forming pores in the membrane, parasporin-1 preferred inducing cell death by an apoptotic pathway with a unique cytotoxic mechanism [65,118]. Interestingly, it was reported parasporin-1 could bind to the receptor Beclin 1 in the cell membrane [121]. In mammals, Beclin 1 exists in human breast epithelial carcinoma cell lines with limited expression, but abundant in normal breast epithelia [122]. It plays a key role in autophagy processes and is crucial in several pathways in all eukaryotic species [123]. The interaction of parasporin-1 with Beclin 1 and its cytotoxicity mechanism is waiting to be elucidated.

#### 4.2.2. Parasporin-2

Parasporin-2 was first discovered in *B. thuringiensis* A1547 (formerly 94-F-45-14), which belongs to *B. thuringiensis* serovar *dakota* (H15) [117,124]. The inactive pro-PS2Aa1 is approximately 37 kDa and has limited similarity to most of the established Cry or Cyt proteins, but shares 23.5% sequence identity with Cry15Aa, which belongs to the MTX-like toxins from *L. sphaericus* [125,126]. Proteinase K cleaves pro-PS2Aa1 into a 30 kDa active form after proteolysis at both N- and C-terminus [118,119,125]. The activated toxin shows high cytotoxicity to MOLT-4, Jurkat (Leukemic T cell), Sawano (Uterus cancel cell), and HepG2 (human hepatocyte cancer cell) with the LC_50_ among 10–40 ng/mL. Early study proposed that parasporin-2 specifically targeted the receptor-like proteins in the membrane and induced cell damage [125]. Later, it was revealed that parasporin-2 could selectively bind to cholesterol in the human cancer cell membrane [119,127]. Experiments have demonstrated that the deletion of cholesterol may relate to a reduction in the efficiency of oligomerization. These results suggested parasporin-2 may be a lipid-raft-targeting toxin and could be transformed into a hydrophobic conformation by oligomerization, which would induce pore formation [127]. Among the candidates of parasporin-2 receptors, parasporin-2 shows a light dependence on cholesterol but an obvious requirement for GPI-anchored proteins, which are primarily located in the cholesterol and sphingolipid-enriched lipid rafts [128,129,130]. It is possible that the glycan region of GPI anchor may also assist parasporin-2 binding to the surface and then assembly into the membrane [130]. Accordingly, a multi-step mechanism model has been put forward: at first, parasporin-2 would bind to the GPI-anchored receptors or other proteins in the membrane; then, when toxin concentrated and oligomerized, transmembrane pores are formed, leading to damage to membrane permeability [130].

#### 4.2.3. Parasporin-3

PS3Aa1 and PS3Ab1 of parasporin-3 are from *B. thuringiensis* stain A1462 (formerly 89-T-26-17) at a molecular weight around 88 kDa [117,131]. When treated with proteinase K, both the 88 kDa proteins were degraded at N terminus to produce the 64 kDa activated forms. These two activated 64 kDa proteins have narrow cytotoxicity spectra towards HL60 and HepG2 and could increase cell membrane permeability of HepG2 [66]. Primary sequence analysis revealed that they were encoded by *orf2a* and *orf2b* genes respectively and shared a high sequence homology of 88%, thus the ORF2a and ORF2b proteins were correspondingly designed as PS3Aa1 and PS3Ab1. It is worth mentioning that, although parasporin-3 has a very low sequence similarity with Cry or Cyt toxins, the five typical conserved block regions of the three-domain Cry toxin exist within this group [66]. Based on this, parasporin-3 probably follows the similar receptor-binding model in target cells [66,118].

#### 4.2.4. Parasporin-4

Parasporin-4 was isolated from the soil isolate *B. thuringiensis* A1470 strain (formerly 89-T-34-22), belonging to the serovar *shandongiensis* [132,133]. When digested with proteinase K, an activated 27 kDa protein was isolated and showed strong cytotoxicity against human cancer cells *in vitro*, such as MOLT-4, CACO-2 (human colon cancer), Sawano, TCS (human uterus cervix cancer), and HL60 cells [132,133,134,135,136,137]. Research has revealed that parasporin-4 nonspecifically binds to the plasma membrane, forming an oligomeric pore complex in target cells. During this process, it shows a cholesterol-independent activity, which is distinct from parasporin-2 that requires cholesterol for the cytotoxic activity [130,138]. On the other hand, PSI-BLAST search results have presented that parasporin-4 shares homologies with Cry15Aa, α-toxin, aerolysin, and ε-toxin at sequence identities of 24%, 15%, 10%, and 21%, respectively. Moreover, a CD spectrum of parasporin-4 revealed that it contains 51% of β-structure. Thus, parasporin-4 could be a unique cholesterol-independent β-PFT, while the identification of specific receptor and its transmembrane mechanism need to be addressed [138].

#### 4.2.5. Other Parasporins

Parasporin-5 from *B. thuringiensis* A1100 strain was discovered by Ekino and Shin [13]. Parasporin-6 (CP84 toxin) was isolated from *B. thuringiensis* strain M019, which exhibits 56.4% sequence similarity to the three-domain Cry toxin. It may have extra peptide regions in the third domain. Parasporin-6 shows preferential cytotoxicity towards HepG2 and HeLa cells, with LC_50_ at 2.3 μg/mL and 7.2 μg/mL, respectively, which are lower than the toxicities of parasporin-1 and parasporin-2 against their corresponding human cancer cells [68,120,125].

### 4.3. Structure of Parasporins and Other Aerolysin Family Members

Since the first structure of proaerolysin toxin was solved in 1994, aerolysin-type toxins have been described as newly discovered category and have become the second largest family of PFTs after Cholesterol Dependent Cytolysins (CDCs) [139,140]. This diverse family originates from Gram-positive and Gram-negative bacteria in plants and eukaryotes, which contains but is not limited to: (1) Aerolysin, from *A. hydrophila* and related *Aeromonas* species; (2) *C. septicum* epsilon-toxin and enterotoxin from *C. perfringens*; (3) Alpha-toxin (α-toxin) from *C. septicum*; (4) Enterolobin from *E. contorisiliquum*; (5) mosquitocidal toxins (MTx) from *L. sphaericus*; (6) anti-cancer parasporin-2 toxin from *B. thuringiensis*; (7) *L. sulphureus* lectin (LSL); and (8) Hydralysins from aquatic animals in the phylum *Cnidaria* [140,141,142]. Aerolysin-type toxins have remarkable sequence identity or structural similarity among their domains, and also act as cytolysins via pore-formation [141].

**Figure 6:**
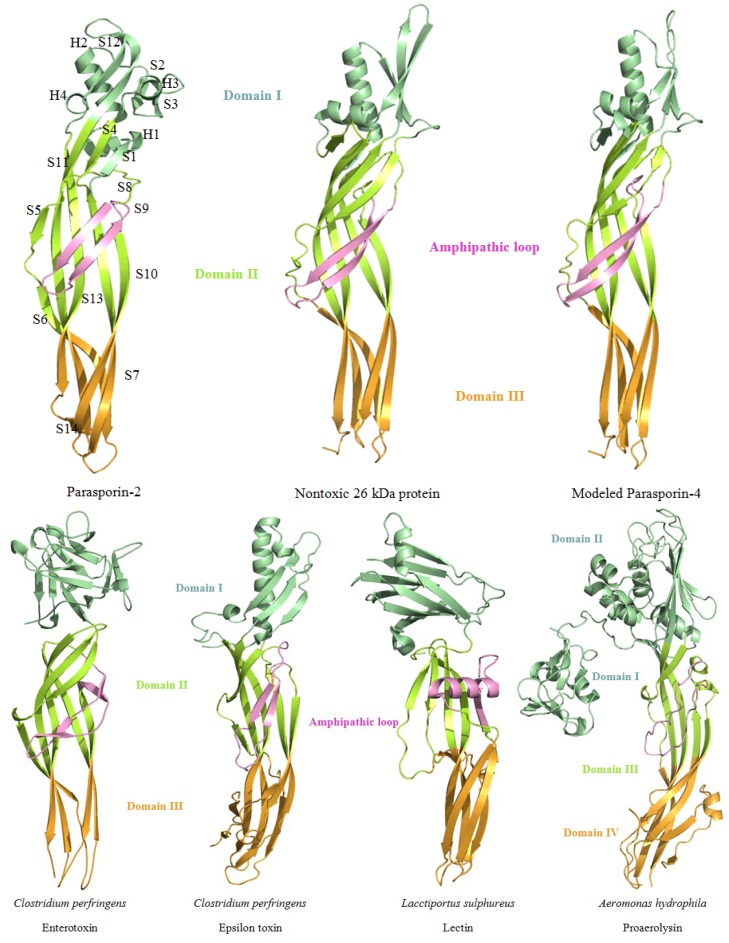
Structural comparison among parasporin-2, the nontoxic 26 kDa protein and the aerolysin-type β-PFTs. The membrane binding related N-domain is colored in pale green. Membrane insertion and pore formation regions are colored in lemon and bright orange, respectively [142]. The pink colored amphipathic β-hairpin is suggested to be responsible for pore formation. Parasporin-4 is modeled using the nontoxic 26 kDa protein as template.

The crystal structures of parasporin-2 (PDB ID 2ZTB) and the nontoxic 26 kDa protein (PDB ID 2D42) were determined recently, the latter of which was a nontoxic protein from *B. thuringiensis* A1470 and shared 38% sequence identity with parasporin-4 [126,143,144]. An automated comparative structure of parasporin-4 is generated by SWISS-MODEL, using the nontoxic 26 kDa protein as template (Figure 6) [145]. A significant structural similarity is found within these structures to the aerolysin-type β-PFTs.

#### 4.3.1. Structure of the Aerolysin-Type Parasporin

##### 4.3.1.1. Domain I of the Aerolysin-Type Parasporin

Domain I was suggested to play an important role in the binding interaction with GPI anchors [146]. The α-helices are existed only in this domain: four short α-helices in parasporin-2 while only two separated α-helices in the nontoxic 26 kDa protein. In domain I, N-terminal strands makes up a short β-hairpin (S2 and S3 in parasporin-2; S9 and S10 in the nontoxic 26 kDa protein) and C terminus includes a part of the longest spanning β-strand (S11 in parasporin-2; S9 in the nontoxic 26 kDa protein). In domain I of aerolysin-type β-PFTs, aromatic residues are abundant. Those aromatic residues could bind to carbohydrates, as galactose and glucose complex: the aromatic rings stack with planar faces of carbohydrate rings via protein-carbohydrate interactions. The interaction between aromatic residues and carbohydrates is determined by the relative position and orientation, which could contribute to the various binding specificity of the sugar-binding proteins [147]. By the structural comparisons, domain I of structural modeled parasporin-4 may have shorter β-strands, which is distinct from the nontoxic 26 kDa protein but similar to parasporin-2. It could be assumed that in the nontoxic 26 kDa protein, the long antiparallel β-strands may protect potential residues from exposure to the solvent, thus blocking the interaction with membrane.

##### 4.3.1.2. Domain II of the β-PFT Type Parasporin

Domain II of parasporin-2 and the nontoxic 26 kDa protein shows highly similarity, which has one β-hairpin and one anti-parallel five-stranded β-sheet [126,143]. In parasporin-2, strand S1 and the five-stranded β-sheet are rearranged to form a four-stranded β-sheet near the boundary to domain I, which associates with the helices of domain I by hydrophobic interactions. Inner surface of the β-sheet and the amphipathic β-hairpin makes up a hydrophobic core, surrounded by the hydrophilic residues on the β-sheet and stabilized by the hydrogen bonds [126]. Arrangement of hydrophobic and hydrophilic residues is a critical feature to direct protein to fold [148]. In the aerolysin-based structural model of α-toxin, the amphipathic β-hairpin could span the membrane and is necessary for pore formation [149]. Also, site-directed mutagenesis in amphipathic β-stand of ETX could alter channel characteristics in lipid bilayer. This suggested that domain II of ETX could be involved in membrane insertion and pore formation [150]. Unlike the nontoxic 26 kDa protein, strands adjacent to the amphipathic β-hairpin in parasporin-2 (strand S4 and S5) and modeled parasporin-4 are separated by loops; it could be deduced that the separated β-strands may be more flexible to facilitate the amphipathic β-hairpin unfold and stretch during the transmembrane process. In addition, parasporin-2 has a remarkable distribution of hydrophobic serine and threonine residues in the β-strands adjacent to the amphipathic β-hairpin in domain II [126]. Similar serine- and threonine-rich flanking sequences are also found in other structural known aerolysin-type β-PFTs, such as ETX, aerolysin, α-toxin, and MTXs [146]. These sequences were proposed to participate in the transmembrane process [126,146].

##### 4.3.1.3. Domain III of the β-PFT Type Parasporin

Domain III of parasporin-2 and the nontoxic 26 kDa protein consists of an anti-parallel three-stranded β-sheet and an anti-parallel two-stranded β-sheet, packed as a β-sandwich, [126,143]. As C-terminal end of parasporin-2 may be removed during proteolytic digestion, some part of the buried hydrophobic core within the β-sandwich is exposed to the solvent, forming a small hydrophobic surface patch along the β-strands. It was suggested that C-terminal activation would be required for oligomerization [126].

#### 4.3.2. Comparisons with Other Structure Known Toxins in Aerolysin Family

Using parasporin-2 as a template, several structures with high similarity are found by DALI server: Z-score of ETX, nontoxic 26 kDa protein, LSL, proaerolysin, and CPE are 10.0, 9.7, 6.5, 6.3 and 5.5, respectively [75,126,139,142,143,146,151]. Based on structural homology and biological relevance, parasporin-2 was proposed as a new member of aerolysin-type β-PFTs [126]. Structural comparisons among aerolysin family members present similarities within domain III and domain IV of aerolysin, domain II and domain III of parasporin-2, ETX, CPE, and LSL, these domains are suggested to be related to oligomerization and responsible for pore formation. Meanwhile, receptor binding related domain I of parasporin-2, ETX, CPE, and LSL and domain II of aerolysin exhibit limited structural similarity.

##### 4.3.2.1. The Most Diverse Domain

Domain I and domain II of aerolysin, and domain I of other members from aerolysin family present the most structural diversity. Differences within these regions probably result in the variation of receptor recognition and toxicity specificity (Table 2). In the newly released structure of CPE, domain I could bind to claudin receptors via the residues exposed on the surface [142,152]. Unlike CPE, domain II of aerolysin monomer may participate in the binding to the GPI-anchored receptors [141,153]. Similar evidence was found in parasporin-2 that GPI-anchored proteins are involved in cytocidal specificity and oligomerization [130]. Although with the most diversity, these regions exhibit several interesting features in common. In aerolysin, aromatic residues are abundant on the surface of domain II, and nearly 70% of the aromatic residues within the whole structure are solvent accessible [139]. Similar aromatic clusters are also found in ETX, LSL and parasporin-2. In parasporin-2, aromatic residues (tryptophan, histidine, phenylalanine, and tyrosine) of domain I display an obvious patch on the surface [126]. The surface-exposed aromatic rings have been recognized as binding determinant to polysaccharides, while this carbohydrate-aromatic stacking is frequently found in carbohydrate-protein complex [154]. In particular, orientation of the aromatic rings may play a major role in specific binding ability [155]. At the N terminus of LSL, aromatic residues (Tyr91/Phe139 and Asp93/Asp141) in the lectin β-trefoil architecture are involved in carbohydrate binding: residues Tyr91 and Phe139 are stacking against the galactose ring of N-acetyllactosamine; side chains of residues Asp93 and Asp141 interact with axial C4 hydroxyl group of galactose by hydrogen bonds [156]. The function of aromatic residues still remains to be demonstrated in parasporin; however, distributions of these surface residues may provide specific interactions with the membrane.

**Table 2:** Characteristics of parasporin proteins and aerolysin-type PFTs *.

Toxins	Strain	PDB ID	Native Size in kDa	Cytotoxicity	Receptor Characteristics	Pore Formation	Oligomerization (number of monomers)	Toxin Type
Parasporin-1	*B. thuringiensis* A1190	unkown	81	Hela, MOLT-4, Hep G2, HL-60	Beclin-1	unknown	unknown	three-domain-type Cry toxin
Parasporin-2	*B. thuringiensis* A1547	2ZTB	37	MOLT-4, Jurkat, Sawano, HL-60, and HepG2	require GPI-anchored protein	possibly	unknown	Aerolysin-type β-PFT
Parasporin-3	*B. thuringiensis* A1462	-	88	HL-60, HepG2	unknown	unknown	unknown	three-domain-type Cry toxin
Parasporin-4	*B. thuringiensis* A1470	-	34	MOLT-4, HL-60, HepH2, Caco-2, Sawano, TCS	cholesterol-dependent	possibly	unknown	cholesterol-independent β-PFT
nontoxic 26 kDa protein	*B. thuringiensis* A1470	2D42	32	nontoxic	unknown	unknown	unknown	nontoxic Aerolysin-type β-PFT
Aerolysin	*Aeromonas hydrophila*	1PRE	52	broad range with GPI-anchored epithelia cells	GPI-anchored receptors	yes	7	Aerolysin-type β-PFT
Epsilon	*Clostridium perfringens*	1UYJ	32.5	limit cell lines as MDCK, G-402	non GPI-anchored membrane protein	yes	7	Aerolysin-type β-PFT
Entertoxin	*Clostridium perfringens*	2XH6	35	intestinal epithelia cells	Claudi	yes	3/6	Aerolysin-typeβ-PFT
LSL	*Laetiportus sulphureus*	1W3A	35	unknown	glycoproteins	possibly	4/6	Aerolysin-type β-PFT

* Table 2 is concluded and modified according to Ref. [118,141].

##### 4.3.2.2. The Pore-Forming Domain

Some features of the pore-forming domain in aerolysin-type PFTs are shared in common. First of all, the amphipathic β-hairpin (or loop) resides in the center of the pore-forming domain. It is plausible that this long β-hairpin spans the lipid bilayer and forms the transmembrane β-barrel in the membrane [157]. When the β-hairpin penetrates the lipid bilayer, as the transmembrane stage take places, the relevant structure would fold or collapse accordingly [152]. Secondly, compared to the surface features of the most diverse domain in the aerolysin family, aromatics residues also cluster at the amphipathic β-hairpin (or loop) and inner surface of the β-sheet [156]. Little is known about these aromatic residues. However, adjacent to the amphipathic β-hairpin (or loop), these residues may take part in the connection with the membrane during the pore formation. In parasporin-2, it was pointed out that the side chain of phenylalanine residue would face towards the membrane and act as an anchor [126]. Thirdly, besides the aromatic residues, serine and threonine residues present a striking distribution on the surface. In ETX, serine and threonine residues are abundant at both sides of the amphipathic loop. It was inferred that serine and threonine residues may be related to the oligomerization [146]. Particularly, it was also believed that after receptor binding but before the pore-forming process, serine and threonine residues may assist the β-hairpin rearrangement and insertion but keep the molecule parallel to the membrane [130]. Last but not the least, like the three-domain Cry toxins, after secretion, the water-soluble native aerolysin-type toxins would turn into activated stage by proteolytic digestion at the sequence terminal. Similar to other toxins, parasporin-2 retains its original tertiary conformation after N- or/and C-terminal digestion. The N terminus of parasporin-2 resides between the amphipathic β-hairpin and the β-strand S4. It was supposed that N terminus may block the transmembrane β-hairpin spanning the membrane, consequently preventing pore formation. The estimated C terminus of parasporin-2 probably consists of a β-strand and α-helix that may protect and cover the exposed hydrophobic part of domain III [126]. In aerolysin, the cleaved C-terminal peptide is not involved in pore formation, but may guide the activated monomer’s rearrangement into oligomers [158,159]. It could be estimated that when C terminus of parasporin is cleaved from the native toxin, the exposed hydrophobic region may facilitate the transmembrane β-hairpin to fold and then insert into the membrane.

##### 4.3.2.3. Membrane Insertion Mechanism

**Figure 7:**
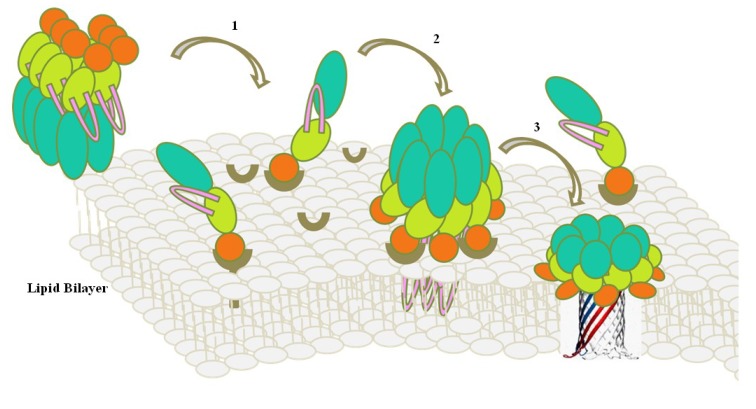
Transmembrane model of aerolysin in lipid bilayer (modified according to Ref [159]). Domain I and domain II of aerolysin are colored in orange; domain III in lemon; domain IV in pale green; and the amphipathic loop in pink. According to the aerolysin swirling model, receptor binding and transmembrane steps are as follows: 1. Solubilized aerolysin binds to the GPI-anchored receptors in the lipid rafts at N-terminus. This process facilitates aerolysin monomer a 180° rotation to make its N-terminal end facing the membrane. 2. After C-terminal proteolytic digestion, the activated aerolysin monomers assemble into a heptamer, as well as the N terminus of aerolysin remains binding to receptors during oligomerization. 3. The amphipathic loop of domain II swirls and rearranges into a transmembrane β-barrel.

In 1994, when the X-ray structure of proaerolysin was published, a pore-forming model of aerolysin was built [139]. It is the first time that the transmembrane model of aerolysin-type PFTs was demonstrated. Seven monomers of activated aerolysin grouped into a heptamer without obvious conformational changes and inserted into the membrane [160]. Then, with the help of Cryo-EM and the three-dimensional reconstruction method, it was observed that an aerolysin mutant Y221G could form a water-soluble mushroom-shaped heptamer. In this model, the whole domain IV spans the membrane as the stem region in the lipid bilayer [161]. Later, a rivet-like model was put forward. It was proposed that the amphipathic β-hairpin could play a role in transmembrane procedure, and penetrate the lipid bilayer as a requirement for membrane insertion but not for heptamerization. The hydrophobic tips of the amphipathic loop would fold back towards the channel, and parallel to the plane of the membrane, like an upside-down rivet [162]. The rivet-like model describes a transmembrane stage of the aerolysin heptamer, which is mainly caused by the reorganized amphipathic β-hairpin. Recently, a new membrane insertion model was presented based on the combined technologies of Cryo-EM, X-ray crystallography, molecular dynamics, and computational modeling [159]. When proaerolysin monomer firstly interacts with the membrane, domain II would bind to the GPI-anchored receptors, indicating domain II is facing the membrane and the entire toxin is 180° rotated. Later, monomers assemble into a heptamer by proteolytic activation at the C terminus of domain IV. During the heptamerization, domain II retains the binding with GPI-anchored receptors. Therefore, the pre-pore structure is achieved by a 180° rotated rearrangement from a mushroom-shape into a disk-like heptamer. In the following transmembrane steps, the amphipathic loop of domain III swirls and rearranges into a β-hairpin, then combines with the neighboring loops of the pore-pore heptamer into a transmembrane β-barrel (Figure 7). The swirling transmembrane model is consistent with the available structural and biochemical data, and is recognized as the actual membrane insertion process [159,163]. This model offers us a beautiful example with key procedures during membrane insertion. Although the fundamental working mechanism and membrane penetration features are varied in aerolysin family members, the elucidated transmembrane models of aerolysin have provided valuable insights that will be explored and identified in other aerolysin-type PFTs in the future.

## 5. Conclusions

Based on known or predicted structures and structure–function relationships, we describe *B. thuringiensis* Cry, Cyt and parasporin toxins into three different categories: three-domain type α-PFTs, Cyt toxin type β-PFTs and aerolysin type β-PFTs. By inter- and inner-comparison among *B. thuringiensis* toxins and PFTs or other structural related protein, an apparent outline of structural and functional features within these three groups has been drawn. Although numerous researches have been dedicated to investigating the role of specific structural components, and many clear interactions between membrane and toxins have been described, the mechanism of action still remains controversial, and there is still a long way to go before it can be elucidated unequivocally. Nevertheless, because of these contributions, applications of *B. thuringiensis* toxin and strategies for overcoming resistance or cross-resistance have developed rapidly.

It is also worth mentioning that some toxins have been suggested with unique toxicities and possible distinct structures. As Cry6A, a nematicidal toxin shows low similarity to the three-domain Cry toxins and is lacking any of the five conserved blocks [32,164]. Cry22A and Cry34/Cry35 binary toxin have also been estimated with diverse structural characteristics [14,165,166]. As the number of discovered toxins rises, and novel structures are being solved, a new member of *B. thuringiensis* toxins would be described under these three categories or even according to other unidentified groups in the future. Comprehensive understanding of current structural related features may deepen the knowledge of novel toxins and accelerate the development of novel products in pest management and human health care.

Moreover, primary sequences and structural similarity have predicted an evolutionary approach within *B. thuringiensis* toxins [14]. On the other hand, evolutionary changes of individual amino acid residues could alter protein binding interactions and secondary elements, resulting in various specific toxicities and different tertiary structures. Upon structural analysis of Cry, Cyt and parasporin toxins, however, it could be hypothesized that an inner-molecular domain shift (e.g., domain I of three-domain Cry toxin and aerolysin-type parasporin) or domain insertion/deletion (e.g., β-sheet of Cyt toxin) may take place during the evolutionary process, which consequently causes structural and functional divergence of *B. thuringiensis* toxins.
